# Development and validation of a headspace GC-MS method to evaluate the interconversion of impurities and the product quality of liquid hand sanitizers

**DOI:** 10.1186/s41120-021-00049-8

**Published:** 2022-01-17

**Authors:** Nicolas Abrigo, Connie Ruzicka, Patrick Faustino, Neil Stiber, Agnes NguyenPho, Thomas O’Connor, Diaa Shakleya

**Affiliations:** 1 Division of Product Quality Research, Office of Testing and Research, Office of Pharmaceutical Quality, U.S. Food and Drug Administration, 10903 New Hampshire Ave., Life Sciences Building 64, Silver Spring, MD 20993 USA; 2 Division of Pharmaceutical Analysis, Office of Testing and Research, Office of Pharmaceutical Quality, U.S. Food and Drug Administration, 645 S Newstead Ave., St. Louis, MO 63110 USA; 3 Division of Quality Intelligence II, Office of Quality Surveillance, Office of Pharmaceutical Quality, Center for Drug Evaluation and Research, U.S. Food and Drug Administration, 10903 New Hampshire Ave., Silver Spring, MD 20993 USA

**Keywords:** Validation, Headspace GC-MS, Alcohol hand sanitizer, COVID-19, Stability

## Abstract

The COVID-19 pandemic has led to increased usage of hand sanitizer products by the public to prevent the spread of COVID-19 and decrease the likelihood of acquiring the disease. The increase in demand has also led to an increase in the number of manufacturers. This work describes the FDA’s Center for Drug Evaluation and Research (CDER) laboratories efforts to develop tests to assess the quality of hand sanitizer products containing ethanol or isopropanol as the primary active ingredient. The products were evaluated for the active ingredient content and determination of the 12 impurities listed in the FDA Hand Sanitizer Temporary Guidance, followed by a spike recovery assay performed to verify the test results. Extensive method development was conducted including an investigation into the stability of ethanol, isopropanol, and the 12 impurities. Stability and kinetic studies confirmed the instability of acetal in acidic liquid hand sanitizer products during spike recovery assay testing. The headspace GC-MS method was validated according to ICH Q2 (R1) guidelines and the spike recovery assay was validated using three concentrations of standards for the drug product. During method application, six liquid hand sanitizer products were tested and all were determined to have ethanol or isopropanol above 70% v/v. Two liquid hand sanitizer products were determined to contain acetaldehyde as an impurity above the FDA recommended safety levels.

**Supplementary Information:**

The online version contains supplementary material available at 10.1186/s41120-021-00049-8.

## Introduction

Responding to the Coronavirus Disease 19 (COVID-19) pandemic, the World Health Organization (WHO) and the Center for Disease Control (CDC) have placed a heightened importance on hand hygiene. The WHO and CDC have advised the use of alcohol-based hand sanitizers to help prevent viral infection when soap and running water were not available (WHO 2010; Singh et al. 2020; Golin et al. 2020; CDC 2020). These recommendations by the WHO and CDC led to a shortage in hand sanitizer supplies in early 2020 prompting the Food and Drug Administration (FDA) to publish a temporary guidance on testing and a temporary guidance on manufacturing quality of hand sanitizer products (FDA 2020a,b). The FDA temporary guidance detailed that finished liquid hand sanitizer products should be formulated to be either 80% ethanol (v/v) or 75% isopropanol (v/v) in an aqueous solution (FDA 2020a), with limits following the CDC recommendation of at least 60% ethanol or 70% isopropanol (CDC 2020). The dramatic increase in demand for ethanol (ethyl alcohol) has resulted in the diversion of alcohols generally used for industrial applications to the manufacturing of hand sanitizer products likely causing contamination with undesirable solvents or impurities.

The FDA has continued to regulate hand sanitizer products to ensure to product quality and protect the public from unsafe products from poor manufacturing practices (FDA 2020c; Chan and Chan 2018; FDA 2020d; FDA 2021). The FDA temporary guidance highlighted 12 impurities that should be monitored in the finished products (FDA 2020a). The allowable concentration limits detailed in the FDA temporary guidance was modified from the ICH Q3C Guideline on Impurities: Guideline for Residual Solvents (ICH 2016) and the USP general chapter <467> Residual Solvents (USP 2020) (Table 1). The solvents were classified based on their toxicities where class 1 solvents should be avoided from use, class 2 solvents should be limited in use, and class 3 solvents contain low toxic potential. Benzene is a class 1 impurity and methanol is a class 2 impurity. Acetone, 1-propanol, ethyl acetate, 2-butanol, Isobutanol, 1-butanol, 3-methyl-1-butanol, and amyl alcohol are all class 3 impurities. According to the updated FDA temporary guidance on the manufacture of alcohol for alcohol based hand sanitizer products, the source of the impurities may be from ethanol manufacturers (FDA 2020a,b) and/or chemical interconversion between these impurities based on the research work reported in this manuscript. Acetaldehyde was included in the list of impurities due to its potential genotoxic and carcinogenic properties when in direct contact with tissues (FDA 2020a). Acetal was included because it is a potential precursor for acetaldehyde (Kresge and Weeks 1984; Liu and Thayumanavan 2017). As of 7 October 2021, the FDA had over 260 entries of hand sanitizer products that the FDA was urging the public to not use for reasons such as being subpotent or having impurities above the FDA temporary guidance concentration limits (FDA n.d.).

**Table 1:** Impurity limits and GC-MS conditions of active ingredients, impurities, and internal standards

Compound	Conc. limit (ppm)	Ret. time (min)	Quantifier ion (m/z)	Qualifier ion (m/z)
Ethanol		1.82	45	31
Isopropanol		2.28	45	29
Acetaldehyde	50	1.28	43	29
Methanol	630	1.34	31	29
Benzene	2	4.13	78.1	51
Acetal	50	4.89	73.1	45.1
Acetone	4400	2.13	58.1	43
1-Propanol	1000	3.12	31.1	59.1
Ethyl Acetate	2200	3.54	61.1	43.1
2-Butanol	6200	3.64	59.1	45.1
Isobutanol	21700	4.01	31.1	43.1
1-Butanol	1000	4.52	56.1	41.1
3-Methyl-1-Butanol	4100	5.38	70.1	55.1
Amyl Alcohol	4100	5.72	70.1	55.1
Acetone-d6		2.10	64.1	46
Cyclohexane		3.93	84.1	56.1

Analytical methods using infrared (IR) and Raman spectroscopy have been developed for determining ethanol and isopropanol content in hand sanitizer products (Fonseca Jr., and Brito LRe, Pimentel MF, Leal LB. 2020; Gupta et al. 2021; Pasquini et al. 2020; Evans and Ariel 2020; ACd et al. n.d.). These spectroscopic techniques are non-destructive and obtain results quickly but they lack the sensitivity required to detect many impurities. The FDA developed an initial gas chromatography mass spectrometry (GC-MS) direct inject method to address concerns related to active ingredient content and impurities in hand sanitizer products (FDA 2020e) and additional application notes by Agilent, Phenomenex, ThermoFisher, and Shimadzu have been reported using GC-FID and GC-MS (Zhang n.d.; Dhandapani 2020; Riccardino 2020; Iwasa 2021).

As the demand for hand sanitizer products increased, many new and non-traditional pharmaceutical manufacturers formulated products that became widely used by the American public to help prevent the spread of COVID-19 infections. Thus, the FDA required a comprehensive analytical method to accurately test hand sanitizer products available in the US marketplace for impurities and alcohol content supporting the Agency’s increased surveillance efforts of domestically manufactured alcohol-based hand sanitizer products. To assist the FDA’s role in regulating hand sanitizer products, an improved GC-MS method based on headspace sampling instead of direct injection was developed to determine the active ingredient content and impurity concentration in hand sanitizer products. The headspace GC-MS analytical procedure was developed and validated according to ICH Q2 (R1) (ICH 2005) for specificity, linearity, range, accuracy, precision, limit of detection (LOD), limit of quantitation (LOQ), robustness, stability, dilution integrity, and spike recovery. During testing of liquid hand sanitizer products, variability in the spike recovery of acetal and acetaldehyde for some products was observed. Importantly, the variability only occurred in products containing ethanol as the active ingredient. The potential for interconversion between acetal and acetaldehyde, which was found to be pH dependent, has been discussed in the scientific literature (Kresge and Weeks 1984; Liu and Thayumanavan 2017). Application of the method was performed using six liquid hand sanitizer products containing ethanol or isopropanol as the active ingredient.

This research describes the development of a headspace GC-MS method for ethanol, isopropanol, and 12 impurities in liquid hand sanitizer products. A unique challenge in the spike recovery of impurities was addressed and the cause of the variability in analytical results was determined to be hand sanitizer products with acidic pH. The headspace GC-MS method was validated according to ICH Q2 (R1) (ICH 2005) specifications and was tested using six liquid hand sanitizer products containing ethanol or isopropanol as the active ingredient. The analytical method will assist the FDA in the regulatory testing of liquid hand sanitizer products to ensure they contain a satisfactory level of active ingredients to protect against COVID-19, as well as to help safeguard that impurities are below the levels listed in the FDA temporary guidance on hand sanitizers (FDA 2020a).

## Materials and methods

### Chemicals and reagents

Ethanol (459828, Lot: SHBL9724), acetaldehyde (00071, Lot: SHBK4140), acetal (55795, Lot: BCCD7490), acetone-d6 (444863, Lot: MKCL5546), cyclohexane (PHR1687, Lot: LRAC0291), dimethyl sulfoxide (D4540, Lot: SHBK6028), and acetic acid (27225, Lot: SZBE0640V) were purchased from Sigma Aldrich. Certified reference standards of isopropanol (Lot: R048P0), methanol (Lot: R084K0), benzene (Lot: R101F0), acetone (Lot: I0M548), 1-propanol (Lot: R04060), ethyl acetate (Lot: R09350), 2-butanol (Lot: R09520), Isobutanol (2-methyl-1-propanol, Lot: R019D0), 1-butanol (Lot: R048N0), 3-methyl-1-butanol (Lot: F0E242), and amyl alcohol (1-pentanol, Lot: F0E214) were purchased from the United States Pharmacopoeia (USP, Rockville, MD). Water (W6-4, Lot: 203956), ammonium hydroxide (A470-250, Lot: 7215090), and ammonium acetate (A114-50, Lot: 156070) were purchased from Fisher Scientific. Dimethyl sulfoxide (1019001000, Lot: I1053100) was purchased from EMD Millipore. Orion buffer standards with pHs of 1.68, 4.01, 7, and 10.01 were purchased from Thermo Scientific. The liquid hand sanitizer products were received through the sampling assignment program issued by the Office of Quality Surveillance, a suboffice in the Center of Drug Evaluation and Research’s (CDER) Office of Pharmaceutical Quality.

## Methods

An Agilent GC 7890A coupled with a headspace sampler 7697A and an MSD 5977B was used for the analysis. A J&W DB-Select 624 UI GC column (Agilent 123-0334UI, SN: US0341521H) was used for analysis. As part of method development, a J&W DB-Wax UI GC Column (Agilent 123-7033UI, SN: US16210002) was also tested. An Agilent ultra-inert split liner (5190-2295, Lot: 90443) was used in the inlet. Headspace GC vials (20 mL, 5190-3986) with Teflon/silicon septa screw caps (5188-2753) were purchased from Agilent. Rainin E4XLS electronic repeater pipettes were used for preparation of standards and samples. A vortex mixer and Mettler Toledo analytical balance were used for preparation of standards and samples. Chemglass amber vials and Pyrex glass bottles were used for storage of standards and samples.

Headspace extraction of the 2 mL samples was performed at 125 °C for 10 min. One milliliter of headspace volume was then injected into the GC column over a period of 1 min using a split ratio of 100:1. The GC oven temperature started at 40 °C for 2 min, then increased at 20 °C/min to 160 °C, then increased at 60 °C/min to 240 °C, and held at 240 °C for 4 min. The GC run time was 13.33 min, post run time was 2 min at 240 °C, and the GC cycle time was 20 min. Mass detection by selected ion monitoring (SIM) was used for identification of product peaks. Retention times, quantifier ions, and qualifier ions for all compounds are listed in Table 1. Three SIM windows were used for detection. SIM window 1 (1-2.9 min, 9 ions) contained acetaldehyde, methanol, ethanol, acetone, acetone-d6 (ISTD), and isopropanol. SIM window 2 (2.9–4.35 min, 9 ions) contained 1-propanol, ethyl acetate, 2-butanol, Isobutanol, cyclohexane (ISTD), and benzene. SIM window 3 (4.35-7 min, 8 ions) contained 1-butanol, acetal, 3-methyl-1-butanol, and amyl alcohol.

### Pre-validation stability experiments

An Oakton series 11 pH meter was used for the pH measurements. The pH meter was calibrated using a 3-point calibration that covered the pH range of 1.68–7 or 4–10. After each measurement, the pH electrode was rinsed with deionized water.

Each pH kinetic study was performed on a separate day. The compounds (acetal, acetaldehyde, or ethanol) were prepared as a concentrated stock solution. Three minutes prior to the 0 h timepoint, 100 μL of the concentrated analyte was added to 9.9 mL of buffer solution and the vial was vortexed for 30 s. Three minutes before each timepoint, a 200-μL aliquot was added to a GC vial containing 1.8 mL DMSO. The GC cycle time was 20 min so timepoints of 0, 1.66, 3.33, and 6 h were selected to accommodate the analysis of the five buffer solutions. Negative controls (no analyte) were prepared by taking a 200-μL aliquot of the buffer only and adding to a GC vial containing 1.8 mL DMSO.

Buffers for kinetic study. A stock solution of 0.2 M ammonium acetate buffer was prepared using 1.54 g of ammonium acetate per 100 mL water. The 0.2 M ammonium acetate buffer had a pH of 6.6. For the ammonium acetate buffers at pH 3 and pH 5, concentrated acetic acid was added dropwise until the desired pH was reached. For the ammonium acetate buffer at pH 8.5, concentrated ammonium hydroxide was added dropwise until the desired pH was reached. In addition to the four ammonium acetate buffers, water was included as a control.

Acetal solution for kinetic study. One hundred twenty-seven microliters of acetal and 9.87 mL of DMSO were combined.

Acetaldehyde solution for kinetic study. One hundred twenty-seven microliters of acetaldehyde and 9.87 mL of DMSO were combined.

Ethanol solution for kinetic study. Pure ethanol was used.

### Method validation

The analytical method was validated according to ICH Q2 (R1) (ICH 2005) for specificity, linearity, range, accuracy, precision, LOD, LOQ, robustness, stability, dilution integrity, and spike recovery. Specificity was evaluated by comparing the peak response of the LLOQ QC to the diluent control and internal standard control. A linear calibration curve was constructed as a least square fit of measured peak areas for known calibration standard concentrations. Linearity was determined by calculating the regression line by the method of least squares with a weighting factor of 1/x. Linearity was established by demonstrating accuracy and precision over the analytical range. Stability was determined in both diluent and hand sanitizer product samples. Spike recovery was validated using three concentrations of standards spiked into hand sanitizer samples and compared to identical spikes in diluent.

### Preparation of solutions (method validation and application)

Individual stock of benzene (428 μg/mL). Four hundred twenty microliters of a 10.2 mg/mL benzene reference standard was added to a 20 mL vial containing 9.58 mL of DMSO.

Individual stock of acetaldehyde (4985 μg/mL). One hundred twenty-seven microliters of an acetaldehyde standard was added to a 20-mL vial containing 19.873 mL of DMSO.

Individual stock of acetal (10554 μg/mL). One hundred twenty-seven microliters of an acetal standard was added to a 20-mL vial containing 9.873 mL of DMSO.

Individual stock of acetone-d6 (30,020 μg/mL). Three hundred eighty microliters of an acetone-d6 standard was added to a 20-mL vial containing 9.62 mL of DMSO.

Individual stock of cyclohexane (10049 μg/mL). One hundred twenty-nine microliters of a cyclohexane standard was added to a 20-mL vial containing 9.871 mL of DMSO.

Mixture A (ethanol and isopropanol). Two milliliters of ethanol and 2 mL of isopropanol were added to a 100 mL bottle containing 36 mL of DMSO.

Mixture B (methanol, benzene, acetaldehyde, acetal). 3.61 mL of a 14.8 mg/mL methanol reference standard, 400 μL of individual stock of benzene, 400 μL of individual stock of acetal, and 852 μL of individual stock of acetaldehyde were added to a 100-mL bottle containing 34.74 mL of DMSO.

Mixture C (acetone, 1-propanol, ethyl acetate, 2-butanol, Isobutanol, 1-butanol, 3-methyl-1-butanol, amyl alcohol). Two hundred thirty-seven microliters of acetone, 53 μL of 1-propanol, 104 μL of ethyl acetate, 328 μL of 2-butanol, 1.15 mL of isobutanol, 52 μL of 1-butanol, 215 μL of 3-methyl-1-butanol, and 215 μL of amyl alcohol were added to a 20-mL vial containing 17.6 mL of DMSO.

System suitability mixture. Two milliliters of mixture A and 2 mL of mixture B were added to a 20-mL vial containing 16 mL of DMSO. The vial was capped and vortexed for 20 s.

Internal standard mixture. One milliliters of individual stock acetone-d6 and 1 mL of individual stock of cyclohexane were added to 250 mL bottle containing 98 mL of DMSO. The bottle was capped and mixed for 30 s.

### System suitability

The system suitability standard containing ethanol, isopropanol, methanol, benzene, acetal, and acetaldehyde was injected six times at the start of each day of sample analysis. The retention time, peak area, USP tailing factor, and resolution were recorded for each peak. The repeatability of retention time (< 2% relative standard deviation (RSD)), peak area (< 2%RSD), USP tailing factor (< 2, < 10%RSD), and resolution (> 2) based on six replicate injections was calculated.

### Calibration standards

Calibration curves (CC) for ethanol, isopropanol, benzene, acetaldehyde, acetal, and methanol were prepared using seven concentration levels. Linearity was determined by calculating the regression line by the method of least squares with a weighting factor of 1/x. Linearity was expressed as the correlation coefficient (r2) and required to be ≥ 0.99.

Preparation of calibration standards (25–400% of the allowable impurity limit). Seven vials were appropriately labeled CC1-CC7. Thirty-eight milliliters of DMSO was added to CC1, 18 mL of DMSO was added to CC2, 8 mL of DMSO was added to CC3, 4.66 mL of DMSO was added to CC4, 3 mL of DMSO was added to CC5, 1.33 mL of DMSO was added to CC6, and 0.5 mL of DMSO was added to CC7. To each CC vial was then added 1 mL of mixture A and 1 mL of mixture B. Each vial was capped and vortexed for 20 s.

### Quality control standards

Quality control (QC) standards (*n* = 3, at each QC level) were prepared using the mixture A and mixture B stock solutions in DMSO. All QC control standards were prepared in triplicate by adding 1.6 mL of DMSO, 0.2 mL QC control standard, and 0.2 mL of internal standard to the headspace GC vial.

Lower limit of quantitation (LLOQ) QC. One hundred fourteen milliliters of DMSO, 3 mL of mixture A, and 3 mL of mixture B were combined and vortexed for 30 s. The LLOQ QC concentration for each impurity was 25% of the allowable limit for hand sanitizer products.

Low QC. Thirty-four milliliters of DMSO, 3 mL of mixture A, and 3 mL of mixture B were combined and vortexed for 30 s. The Low QC concentration for each impurity was 75% of the allowable limit for hand sanitizer products.

Mid QC. Fourteen milliliters of DMSO, 3 mL of mixture A, and 3 mL of mixture B were combined and vortexed for 30 s. The Mid QC concentration for each impurity was 150% of the allowable limit for hand sanitizer products.

High QC. Four milliliters of DMSO, 3 mL of mixture A, and 3 mL of mixture B were combined and vortexed for 30 s. The high QC concentration for each impurity was 300% of the allowable limit for hand sanitizer products.

### Spike mixtures

Spike mixture at 50% of the allowable impurity limit. Ninety milliliters of DMSO, 5 mL of mixture A, and 5 mL of mixture B were combined and vortexed for 30 s.

Spike mixture at 100% of the allowable impurity limit. Thirty-five milliliters of DMSO, 5 mL of mixture A, 5 mL of mixture B, and 5 mL of mixture C were combined and vortexed for 30 s.

Spike mixture at 200% of the allowable impurity limit. Fifteen milliliters of DMSO, 5 mL of mixture A, and 5 mL of mixture B were combined and vortexed for 30 s.

### Hand sanitizer sample preparation

The pH of all hand sanitizer products was measured using an Oakton series 11 pH meter. The density (g/mL) of each hand sanitizer product was determined by adding 2 mL of product to a vial and recording the weight (g).

Preparation of a product for content assay. Thirty-one milliliters of DMSO and 1 mL of hand sanitizer product were combined and vortexed for 30 s. 0.5 mL of the solution was then added to a vial containing 24.5 mL of DMSO and vortexed for 30 s. To a 20-mL headspace GC vial, 1.6 mL of the hand sanitizer sample in DMSO was added with 0.2 mL of internal standard mixture and 0.2 mL of spike mixture. Negative control samples used 0.2 mL DMSO instead of 0.2 mL spike mixture.

Preparation of a product for impurity analysis. Hand sanitizer volumes were adjusted so that the final concentration of hand sanitizer product in the GC vial was 21.25 mg/mL.

A hand sanitizer product with a density of 0.85 g/mL was prepared by combining 1 mL of hand sanitizer product and 31 mL of DMSO in a vial and vortexed for 30 s. To a 20-mL headspace GC vial, 1.6 mL of the hand sanitizer sample in DMSO was added with 0.2 mL of internal standard mixture and 0.2 mL of spike mixture. Negative control samples used 0.2 mL DMSO instead of 0.2 mL spike mixture.

### Method application

The method was applied to test six liquid hand sanitizer products. The products were tested for active ingredient content and impurity concentration. The system suitability test and calibration curve were prepared as described in the method validation section. Quality control standards at LLOQ QC and high QC concentrations were used to monitor instrument performance during the testing day. Each QC concentration was prepared as *n* = 9 samples. The QC samples were injected in triplicate before the hand sanitizer samples, in the middle of the batch sequence, and after all hand sanitizer samples were injected. The spike recovery assay was conducted using the spike mixture at 100% of the allowable impurity limit.

## Results and discussion

### Optimization of analytical method

The analytical method was designed to quantitate ethanol, isopropanol, methanol, benzene, acetaldehyde, and acetal levels in hand sanitizer products, while also qualitatively determining whether the remaining eight impurities were above or below the allowable impurity limit (Table 1). Injection of samples using static headspace provided cleaner extracts than direct injection of analytes from the complex formulation matrices found in hand sanitizer products (Sparkman et al. 2011). The static headspace technique only uses the gas phases above the sample for injection into the GC column. Initially, two GC columns were evaluated during method development. Based on inert phase interaction with polar solvents, the first column selected was a J&W DB-Wax UI GC column. A variety of initial GC oven temperatures and gradients were tested but ethanol, isopropanol, and methanol retention times were not resolved. The adequate resolution of ethanol, isopropanol, and methanol was important because they share common ions using mass spectrometry detection. For enhanced analyte separation and adequate resolution between compounds, the second column tested was a J&W DB-Select 624 UI GC column. The DB-Select 624 UI was designed specifically for the separation of residual solvents listed in USP <467>. The DB-Select 624 UI provided the necessary separation of ethanol, isopropanol, and the 12 impurities listed in the FDA temporary hand sanitizer guidance (FDA 2020a) and was used for analytical method validation and product testing.

The diluent selection was examined to best accommodate the mixture of alcohols and organics that needed to be evaluated. A mixture of water and DMSO was explored initially but an instability of response was observed using the headspace injection method (Supplemental information Table 1). The detector response of all compounds decreased over time for all DMSO/water mixtures. When a 100% DMSO solution was used, the detector response for all compounds remained stable over multiple days. All stock solutions and preparation of samples were prepared using DMSO only.

### Pre-validation stability experiments

A spike recovery assay was performed to verify the concentration of active ingredient or impurity present in each sample. The assay entailed spiking a known amount of compound into a sample matrix and comparing the response to an identical spike in the diluent. Testing of liquid hand sanitizer products revealed variability of acetal and acetaldehyde recovery during the spike assay in some products. Further investigation revealed that this only occurred with ethanol-based products and that the variability only occurred with acetal and acetaldehyde.

An example of the variability is displayed in Supplementary information Table 2 where three products were selected for spike recovery assay of the 12 impurities. Two of the products displayed variability of acetal and acetaldehyde that were outside the acceptable range of 80–120% recovery. All remaining compounds displayed no variability of recovery during the spike study.

**Fig. 1:**
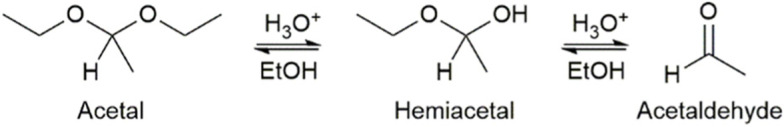
Reaction linking acetal and acetaldehyde

An investigation into the relationship of acetal and acetaldehyde with ethanol was conducted. Acetal, acetaldehyde, and ethanol are intertwined in a pH dependent mechanism (Kresge and Weeks 1984; Liu and Thayumanavan 2017) (Fig. 1). Acidic conditions favor the formation of acetaldehyde while basic conditions favor the formation of acetal. The pH of thirteen liquid hand sanitizer samples was measured and resulted in a range of pH 3.1–8.4 (Supplementary information Table 3). Importantly, the two products that displayed variability in recovery of acetal and acetaldehyde in Supplementary information Table 2, contained acidic measurements of pH 3.1 and 4.6. The reaction in Fig. 1 outlines the conversion of acetal into acetaldehyde under acidic conditions, with a hemiacetal intermediate and ethanol byproduct.

**Fig. 2:**
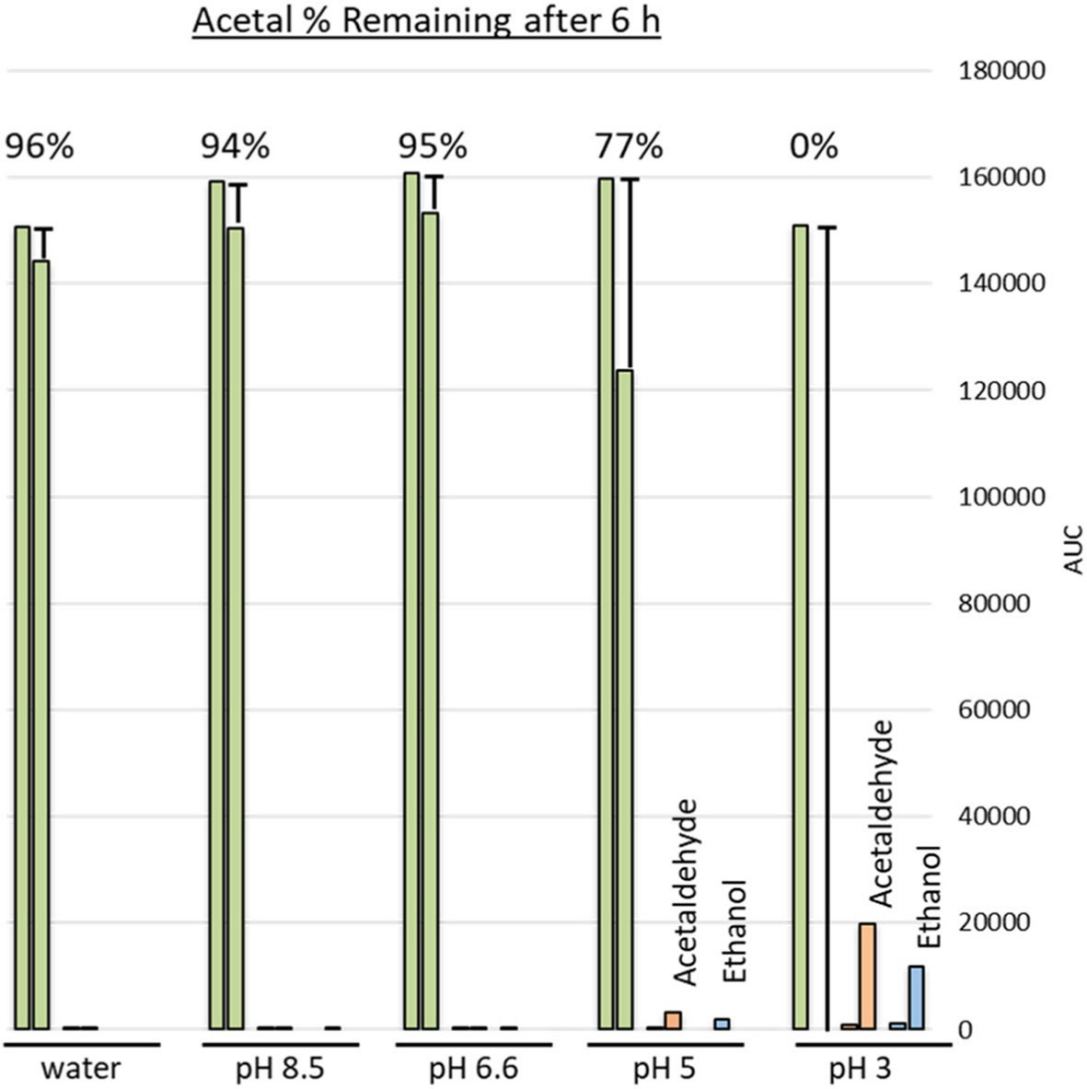
Acetal unstable at pH < 5 during kinetic study*. *Acetal response is depicted in green at 0 h and 6 h. At pH 5 and pH 3, acetal response decreases rapidly and the formation of acetaldehyde (orange) and ethanol (blue) is observed. Acetal is stable in buffers at pH 6.6–8.5

A pH kinetic study was performed to examine the stability of acetal, acetaldehyde, and ethanol at different pHs. The stability of each compound was individually examined over 6 h in 0.2 M ammonium acetate buffers with pH 3–8.5. Water was used as a non-buffer control. Acetal displayed instability at pH ≤ 5 with 0% of acetal remaining after 6 h in the pH 3 buffer (Fig. 2). After 1.66 h at pH 3, only 6% of acetal remained (Supplementary information Table 4). The loss of acetal correlated with formation of acetaldehyde and ethanol. Both acetaldehyde and ethanol displayed stability over the entire pH range tested (Supplementary information Tables 5 and 6).

The pH kinetic studies further confirmed the relationship between acetal, acetaldehyde, and ethanol and their interconversion under acidic conditions. To mitigate the variability of acetal and acetaldehyde recovery, ammonium hydroxide was tested as a neutralizing agent for acidic products. The use of ammonium hydroxide to adjust an ethanol-based liquid hand sanitizer product with pH of 4.6 to a pH of 6 significantly improved the spike recovery of both acetal and acetaldehyde (Supplementary information Table 7).

The pH kinetic studies concluded that the variability in spike recovery of acetal and acetaldehyde impurities was due to the acidity of some liquid hand sanitizer products. The protocol developed for testing hand sanitizer products included the measurement of the pH of the products in addition to the testing for active ingredient content and presence of impurities to ensure accurate testing of all analytes.

### Method validation

The headspace GC-MS analytical method for liquid hand sanitizer products was validated per the requirements from ICH Q2 (R1) (ICH 2005) for specificity, linearity, range, accuracy, precision, limit of detection, limit of quantitation, robustness, stability, and spike recovery. The spike recovery assay in ethanol and isopropanol-based hand sanitizers was validated using three spike concentrations of standards. The method was validated over 3 days by two different analysts.

### System suitability results

The system suitability test measured the performance of the analytical system and must adhere to the criteria stated in USP general chapter <621> chromatography (USP 2017) for retention time, peak area, USP tailing, and peak resolution. The system suitability tests met the acceptable USP criteria during validation and application (Table 2).

**Table 2:** System suitability testing specifications and results

		Retention Time		Area	USP tailing factor		Resolution
Compound	Day	(min)	RSD < 2.0%	RSD < 5%	< 2.0	RSD < 10.0%	> 2.0
Acetaldehyde	1	1.28	0.01	2.67	0.96	1.28	> 2.0
	2	1.28	0.01	3.08	0.98	1.25	> 2.0
	3	1.28	0.01	1.41	0.98	1.50	> 2.0
Methanol	1	1.33	0.01	1.45	1.44	2.14	> 2.0
	2	1.33	0.01	1.74	1.50	1.68	> 2.0
	3	1.33	0.01	2.05	1.50	2.43	> 2.0
Ethanol	1	1.81	0.00	1.39	1.03	2.28	> 2.0
	2	1.81	0.00	1.82	1.07	2.59	> 2.0
	3	1.81	0.00	1.65	0.99	3.20	> 2.0
Isopropanol	1	2.26	0.00	1.43	0.94	1.08	> 2.0
	2	2.26	0.00	1.75	0.95	1.39	> 2.0
	3	2.26	0.00	1.56	0.89	1.91	> 2.0
Benzene	1	4.13	0.00	1.43	0.76	0.40	> 2.0
	2	4.13	0.00	1.47	0.78	1.30	> 2.0
	3	4.13	0.00	2.44	0.77	1.82	> 2.0
Acetal	1	4.88	0.01	2.20	1.00	2.65	> 2.0
	2	4.88	0.01	2.60	1.01	4.43	> 2.0
	3	4.88	0.00	1.24	0.97	1.85	> 2.0

### Specificity

Specificity of the method was determined by observing that no interfering peaks were present in the GC chromatograms of blanks and standards. Compounds were chromatographically separated, identified by retention time, and selectively resolved using MS selective ion monitoring detection with unique quantifier and qualifier ions (Supplementary information Figure 1).

### Linearity and range of calibration curves

The linearity and analytical range for each compound was established using seven standard concentration levels. Ethanol and isopropanol were prepared using a range of 98–1570 μg mL^−1^. Benzene, acetaldehyde, acetal, and methanol were prepared using a range that covered 25–400% of the impurity limits provided in the FDA temporary guidance on hand sanitizers (FDA 2020a). All compounds demonstrated linearity with correlation coefficients *r*^2^ > 0.99 (Table 3).

**Table 3:** Linearity and range data

Analytical range (μg/mL)		Equation	*r*^2^
Ethanol (98.6–1578)	Day 1	*y* = 0.007237*x + 0.115075	0.9961
	Day 2	*y* = 0.007259*x + 0.102598	0.9956
	Day 3	*y* = 0.006469*x + 0.136147	0.9978
Isopropanol (98.1–1570)	Day 1	*y* = 0.026679*x + 0.879872	0.9926
	Day 2	*y* = 0.026658*x + 0.809167	0.9932
	Day 3	*y* = 0.024935*x + 0.805491	0.9949
Benzene (0.0107–0.171)	Day 1	*y* = 0.091878*x − 0.000072	0.9945
	Day 2	*y* = 0.095407*x−0.000148	0.9950
	Day 3	*y* = 0.127246*x + 0.000073	0.9997
Acetaldehyde (0.265–4.25)	Day 1	*y* = 0.026885*x + 0.000006	0.9972
	Day 2	*y* = 0.026836*x − 0.000426	0.9951
	Day 3	*y* = 0.025028*x + 0.000546	0.9991
Acetal (0.264–4.22)	Day 1	*y* = 0.031232*x − 0.002264	0.9931
	Day 2	*y* = 0.034845*x−0.002868	0.9916
	Day 3	*y* = 0.043868*x − 0.001841	0.9992
Methanol (3.34–53.4)	Day 1	*y* = 0.009561*x + 0.005666	0.9960
	Day 2	*y* = 0.009483*x + 0.004019	0.9954
	Day 3	*y* = 0.008795*x + 0.001703	0.9996

### Accuracy

Standards at four QC concentrations were used to determine the accuracy of the analytical method. Results for the intraday accuracy of ethanol, isopropanol, benzene, acetaldehyde, acetal, and methanol are summarized in Supplementary information Table 8. The accuracy for ethanol was 92–109%, for isopropanol was 87–112%, for benzene was 95–108%, for acetaldehyde was 92–110%, for acetal was 94–115%, and for methanol was 99–111%.

### Precision

Standards at four QC concentrations were used to determine the precision of the analytical method. Results for the intraday and interday precision of ethanol, isopropanol, benzene, acetaldehyde, acetal, and methanol are summarized in Supplementary information Table 9. All compounds displayed acceptable precision with %RSD < 5%. Intermediate precision was demonstrated using two different analysts and three separate days of validation.

### Detection and quantitation limits

All compounds displayed a signal-to-noise ratio > 10. The Supplementary information Table 10 displays the S/N at QC LLOQ concentration levels for ethanol, isopropanol, benzene, acetaldehyde, acetal, and methanol.

### Robustness

Robustness of the method was evaluated by making small adjustments in the method parameters. Adjustments were made to the split flow, headspace equilibration temperature, headspace equilibration time, and GC oven temperatures. Each adjustment was evaluated in triplicate using the system suitability mixture. Responses were evaluated for retention time, peak area, USP peak tailing, resolution, capacity factor, and USP theoretical plates. The adjusted methods did not cause a significant change in the response (Supplementary information Table 11).

### Stability

The stock solution stability and autosampler stability in diluent were established over 72 h for all compounds (Supplementary information Tables 12 and 13). The autosampler stability in hand sanitizer products was also established over 72 h for all compounds at concentrations of 25%, 100%, and 300% of the FDA temporary guidance concentration limits (Supplementary information Tables 14, 15, 16).

### Dilution integrity

The active ingredient content assay requires a 2000-fold dilution of the hand sanitizer product in DMSO to bring the ethanol and isopropanol concentrations within the range of linearity. A 70% (v/v) solution of ethanol in water and a 70% (v/v) solution of isopropanol in water were prepared. The dilution integrity test was conducted by diluting the 70% solutions according to the content assay protocol and comparing the determined concentration to the nominal concentration. The ethanol solution displayed accuracy of 108% with a %RSD of 1.9% (Supplementary information Table 17). The isopropanol solution displayed accuracy of 106% with a %RSD of 2.4%.

### Spike recovery assay

The spike recovery assay was validated using two ethanol-based products and two isopropanol-based products. The determined content of active ingredients is reported in Table 4. Spike mixtures were prepared at 50%, 100%, and 200% of the allowable impurity limit provided in the FDA temporary guidance on hand sanitizers (FDA 2020a). The accuracy of recovery was determined by comparing the spiked amount in hand sanitizer sample to the spiked amount in the diluent. The precision was determined by comparing the %RSD of samples examined in triplicate. The results of the spike recovery assay are presented in Tables 5 and 6. The spike recovery for ethanol, isopropanol, benzene, acetaldehyde, acetal, and methanol was performed using all three spike mixtures. A spike recovery for the remaining eight impurities was performed with the 100% spike mixture only.

**Table 4:** Validation of content determination

	Product A	Product B	Product C	Product D
Active ingredient	Ethanol	Ethanol	Isopropanol	Isopropanol
Determined content % (v/v)	80.6%	86.9%	84.4%	85.7%
Impurities above limit	Acetaldehyde 78 ppm	Acetaldehyde 177 ppm	None	None
		Acetal 200 ppm

**Table 5:** Validation of spike recovery assay for active ingredients and level 1 impurities using concentrations at 50%, 100%, and 200% of the temporary guidance limits

	**Product A**			**Product B**		
	Spike mixture 50%	Spike mixture 100%	Spike mixture 200%	Spike mixture 50%	Spike mixture 100%	Spike mixture 200%
Ethanol	95	90	94	106	100	91
Isopropanol	104	97	101	105	103	99
Benzene	85	86	88	93	97	96
Acetaldehyde	88	94	96	101	94	95
Acetal	84	90	92	105	90	104
Methanol	92	99	97	89	96	94
	**Product C**			**Product D**		
	Spike mixture 50%	Spike mixture 100%	Spike mixture 200%	Spike mixture 50%	Spike mixture 100%	Spike mixture 200%
Ethanol	102	98	103	103	101	100
Isopropanol	93	86	94	93	92	89
Benzene	107	106	104	100	100	102
Acetaldehyde	94	95	97	94	97	97
Acetal	108	105	108	110	107	109
Methanol	97	100	100	96	98	99

**Table 6:** Validation of spike recovery assay for level 2 impurities using concentration at 100% of the temporary guidance limits

	Product A	Product B	Product C	Product D
	Spike mixture 100%	Spike mixture 100%	Spike mixture 100%	Spike mixture 100%
1-Propanol	114	113	108	104
1-Butanol	112	111	105	102
Ethyl Acetate	111	110	102	99
3-Methyl-1-Butanol	109	108	105	104
Amyl Alcohol	107	108	104	102
Acetone	104	101	99	98
2-Butanol	113	112	105	102
Isobutanol	112	110	103	100

### Method application

The analytical method was used to test six hand sanitizer products. Product A from method validation was included in the method application, along with 5 different products that were labelled as products E–I. During testing, check QC standards were prepared at QC LLOQ and QC High concentrations to monitor instrument performance for accuracy and precision. The QC standards were injected in sets of triplicates at the start, middle, and end of product testing. The accuracy and precision of the QC standards were evaluated as individual sets (*n* = 3) and as a collective group (*n* = 9). The accuracy among all compounds ranged from 90 to 110% and the precision ranged from 0.3 to 5.7%RSD (Supplementary information Table 18). The hand sanitizer products were tested for active ingredient content and for impurity concentration. A spike recovery assay was performed using the 100% spike mixture (Supplementary information Table 19).

**Fig. 3:**
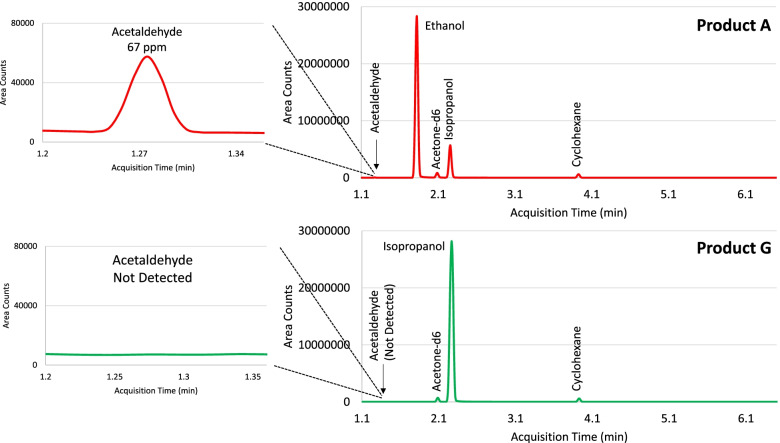
Chromatogram of product A with acetaldehyde detected above the concentration limit and product G with no acetaldehyde detected*. *The top chromatograms in red represent product A and the bottom chromatograms in green represent product G. The chromatograms on the left are scaled so that the acetaldehyde impurity could be visualized. The chromatograms on the right are scaled to the peak of the active ingredients

Following product testing, two of the hand sanitizer products were determined to be ethanol-based and four of the products were determined to be isopropanol-based (Table 7). The active ingredient content ranged from 71 to 88% among all six products. Acetaldehyde was detected at 99 ppm and 67 ppm in two products, which is above the 50 ppm allowable limit stated in the FDA temporary guidance on hand sanitizers (FDA 2020a). Both products containing acetaldehyde above the allowable limit were ethanol-based. One of the isopropanol products contained methanol at 486 ppm, but this was below the 630 ppm allowable limit. Figure 3 provides chromatograms of product A with acetaldehyde impurity detected above the concentration limit and product G with no detection of acetaldehyde impurity.

**Table 7:** Hand sanitizer product testing results

		**Product A**	**Product E**	**Product F**	**Product G**	**Product H**	**Product I**
	Active Ingredient	Ethanol	Ethanol	Isopropanol	Isopropanol	Isopropanol	Isopropanol
	Determined Content (%, v/v)	71.5	86.7	87.7	76.8	80.4	83.9
	pH	7.8	8.3	7.4	8.4	6.6	7.5
**Limit (ppm)**	**Impurity**	**Product A**	**Product E**	**Product F**	**Product G**	**Product H**	**Product I**
2	Benzene	ND	<LLOQ	ND	<LLOQ	ND	ND
50	Acetaldehyde	67	99	<LLOQ	ND	<LLOQ	<LLOQ
50	Acetal	<LLOQ	<LLOQ	<LLOQ	ND	ND	ND
630	Methanol	<LLOQ	<LLOQ	<LLOQ	<LLOQ	<LLOQ	486
1000	1-Propanol	<Limit	<Limit	<Limit	ND	<Limit	<Limit
1000	1-Butanol	ND	ND	ND	ND	ND	ND
2200	Ethyl Acetate	ND	ND	ND	ND	ND	ND
4100	3-Methyl-1-Butanol	<Limit	<Limit	<Limit	<Limit	<Limit	<Limit
4100	Amyl Alcohol	ND	ND	ND	<Limit	ND	<Limit
4400	Acetone	<Limit	ND	<Limit	<Limit	<Limit	<Limit
6200	2-Butanol	ND	ND	ND	ND	ND	ND
21700	Isobutanol	ND	ND	ND	ND	ND	ND

Acronyms in table: *<LLOQ* = detected below LLOQ, *ND* = not detected, *<Limit* = detected below the temporary guidance limit

## Conclusions

In the absence of soap and water, liquid hand sanitizers have been recommended by WHO and CDC to prevent the spread of COVID-19. The developed headspace GC-MS method provided the capability to accurately evaluate ethanol and isopropanol-based products for the active ingredient content and their impurity concentrations. Kinetic studies of pH for acetal and acetaldehyde provided supportive data indicating that the acidic pH of hand sanitizer products caused the acetal and acetaldehyde variability during the spike recovery assay. The method was validated and successfully applied to test six liquid hand sanitizer products for active ingredient content and their impurity concentration. Two of the tested products contained acetaldehyde above the allowable limit stated in the FDA temporary guidance on hand sanitizers, while the remaining 11 impurities were measured and determined to be below the allowable limit in all products. The validated headspace GC-MS method will be applied to liquid hand sanitizer products for determination of active ingredient content and evaluation of all 12 impurities listed in the FDA temporary guidance. The analytical method will assist the FDA in its regulatory actions to ensure the safety and efficacy of hand sanitizer products.

## Supplementary Information

**Additional file 1: Supplementary information Figure 1.** Chromatographic separation of active ingredients, impurities, and internal standards. **Supplementary information Table 1.** Stability of compounds in 100% DMSO solvent. All compounds displayed stability over 18 h in 100% DMSO while response decreased over time when using mixture containing water. **Supplementary information Table 2.** Variability of Acetaldehyde and Acetal During Spike Recovery. The spike recovery assay of Product J and Product K resulted in variability of recovery for acetaldehyde and acetal. Product E displayed recovery of all compounds within the 80-120% specification. **Supplementary information Table 3.** Measured pH of Liquid Hand Sanitizer Products. **Supplementary information Table 4.** Acetal Kinetics Study (% Acetal remaining). The table reports the % acetal remaining over 6 h in the various buffers tested. Acetal displayed instability in pH 5 and pH 3. **Supplementary information Table 5.** Acetaldehyde Kinetics Study (% Acetaldehyde remaining). The table reports the % acetaldehyde remaining over 6 h in the various buffers tested. Acetaldehyde displayed stability in all buffers tested. **Supplementary information Table 6.** Ethanol Kinetics Study (% Ethanol remaining). The table reports the % ethanol remaining over 6 h in various buffers tested. Ethanol displayed stability in all buffers tested. **Supplementary information Table 7.** Spike Recovery after pH Neutralization of Acidic Liquid Hand Sanitizer Product. The recovery of acetaldehyde and acetal is improved after the pH of Product K is adjusted to pH 6 using ammonium hydroxide. **Supplementary information Table 8.** Intraday (n=3) and interday (n=9) accuracy of quality control standards. **Supplementary information Table 9.** Intraday (n=3) and interday (n=9) precision of quality control standards. **Supplementary information Table 10.** Signal-to-Noise at LLOQ. **Supplementary information Table 11.** Robustness Results. **Supplementary information Table 12.** Stock solution stability, 96 h. **Supplementary information Table 13.** Autosampler stability in DMSO, 72 h. **Supplementary information Table 14.** Autosampler Stability in Hand Sanitizer Products, QC LLOQ (25% of NMT Limit), 72 h. **Supplementary information Table 15.** Autosampler Stability in Hand Sanitizer Products, Spiking Solution (100% of NMT Limit), 72 h. **Supplementary information Table 16.** Autosampler Stability in Hand Sanitizer Products, QC High (300% of NMT Limit), 72 h. **Supplementary information Table 17.** Dilution Integrity. **Supplementary information Table 18.** Check QC standards to monitor instrument performance at QC LLOQ and QC High for accuracy and precision during product testing. **Supplementary information Table 19.** Spike Recovery Results During Method Application.

## Data Availability

Not applicable.
