# Supplementary material for: Development and validation of a headspace GC-MS method to evaluate the interconversion of impurities and the product quality of liquid hand sanitizers
Source: AAPS Open. 2022 Jan 17;8(1):1. doi: 10.1186/s41120-021-00049-8 (PMC8762429; doi:10.1186/s41120-021-00049-8)
Supplement: Supplementary file 1 — Additional file 1: Supplementary information Figure 1. Chromatographic separation of active ingredients, impurities, and internal standards. Supplementary information Table 1. Stability of compounds in 100% DMSO solvent. All compounds displayed stability over 18 h in 100% DMSO while response decreased over time when using mixture containing water. Supplementary information Table 2. Variability of Acetaldehyde and Acetal During Spike Recovery. The spike recovery assay of Product J and Product K resulted in variability of recovery for acetaldehyde and acetal. Product E displayed recovery of all compounds within the 80-120% specification. Supplementary information Table 3. Measured pH of Liquid Hand Sanitizer Products. Supplementary information Table 4. Acetal Kinetics Study (% Acetal remaining). The table reports the % acetal remaining over 6 h in the various buffers tested. Acetal displayed instability in pH 5 and pH 3. Supplementary information Table 5. Acetaldehyde Kinetics Study (% Acetaldehyde remaining). The table reports the % acetaldehyde remaining over 6 h in the various buffers tested. Acetaldehyde displayed stability in all buffers tested. Supplementary information Table 6. Ethanol Kinetics Study (% Ethanol remaining). The table reports the % ethanol remaining over 6 h in various buffers tested. Ethanol displayed stability in all buffers tested. Supplementary information Table 7. Spike Recovery after pH Neutralization of Acidic Liquid Hand Sanitizer Product. The recovery of acetaldehyde and acetal is improved after the pH of Product K is adjusted to pH 6 using ammonium hydroxide. Supplementary information Table 8. Intraday (n=3) and interday (n=9) accuracy of quality control standards. Supplementary information Table 9. Intraday (n=3) and interday (n=9) precision of quality control standards. Supplementary information Table 10. Signal-to-Noise at LLOQ. Supplementary information Table 11. Robustness Results. Supplementary information Table 12. Stock solution [file 41120_2021_49_MOESM1_ESM.docx]

**Supplemental Information**

**Development and validation of a headspace GC-MS method to evaluate the interconversion of impurities and the product quality of liquid hand sanitizers**

SI Figure 1: Chromatographic separation of active ingredients, impurities, and internal standards


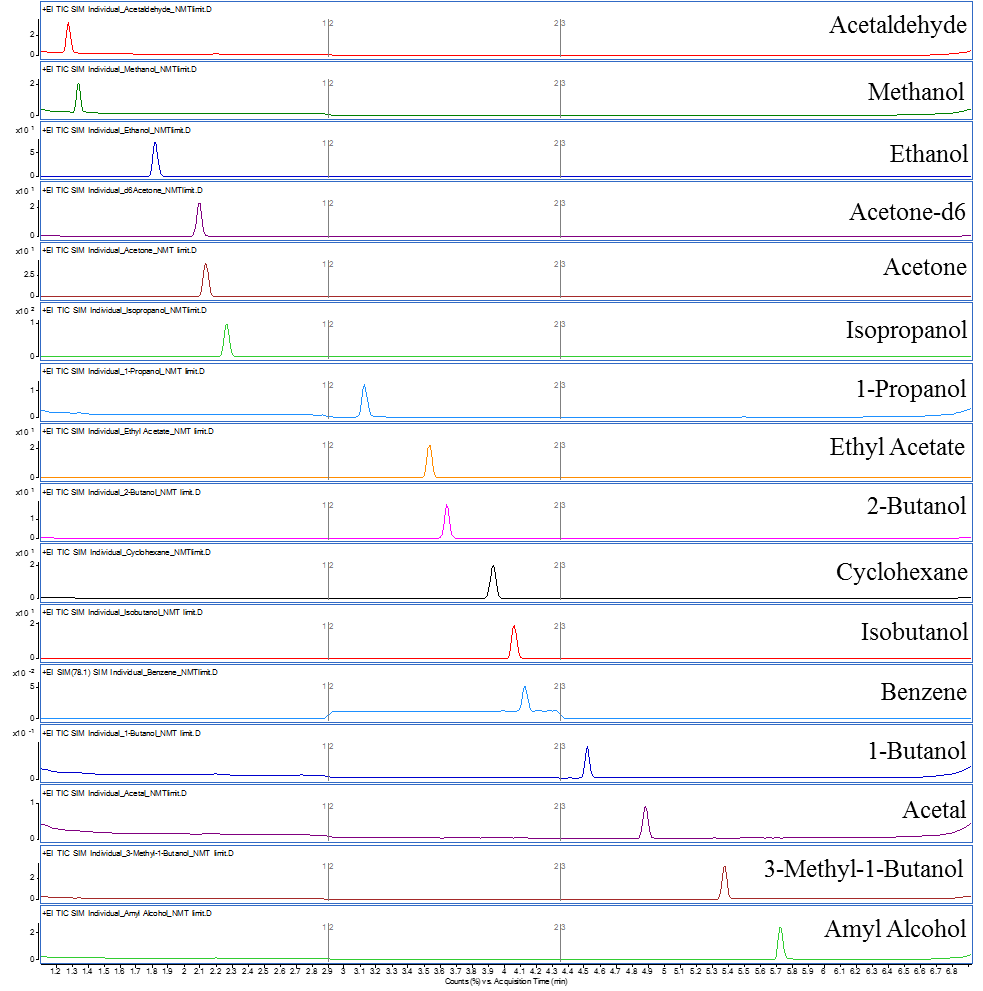


SI Table 1: Stability of compounds in 100% DMSO solvent

All compounds displayed stability over 18 h in 100% DMSO while response decreased over time when using mixture containing water.

| Time (h) | 16 | 16 | 16 | 18 |
| --- | --- | --- | --- | --- |
| DMSO%, Water% | 50%, 50% | 60%, 40% | 70%, 30% | 100%, 0% |
| Ethanol | 76 | 77 | 77 | 97 |
| Isopropanol | 74 | 75 | 76 | 97 |
| Acetaldehyde | 76 | 69 | 75 | 100 |
| Methanol | 75 | 76 | 78 | 98 |
| Benzene | 78 | 80 | 77 | 98 |
| Acetal | 77 | 79 | 76 | 99 |
| Acetone | 78 | 79 | 77 | 98 |
| 1-Propanol | 73 | 76 | 78 | 98 |
| Ethyl Acetate | 76 | 78 | 76 | 98 |
| 2-Butanol | 77 | 78 | 78 | 98 |
| Isobutanol | 77 | 78 | 78 | 98 |
| 1-Butanol | 78 | 75 | 76 | 100 |
| 3-Methyl-1-Butanol | 80 | 77 | 79 | 98 |
| Amyl Alcohol | 77 | 76 | 79 | 98 |

SI Table 2: Variability of Acetaldehyde and Acetal During Spike Recovery

The spike recovery assay of Product J and Product K resulted in variability of recovery for acetaldehyde and acetal. Product E displayed recovery of all compounds within the 80-120% specification.

|  | DMSO Control | Product J | Product K | Product E |
| --- | --- | --- | --- | --- |
| Isopropanol | 100 | 108 | 106 | 106 |
| **Acetaldehyde** | 100 | **47** | **144** | 113 |
| Methanol | 100 | 103 | 104 | 103 |
| Benzene | 100 | 105 | 105 | 105 |
| **Acetal** | 100 | 89 | **38** | 108 |
| Acetone | 100 | 108 | 108 | 106 |
| 1-Propanol | 100 | 109 | 109 | 105 |
| Ethyl Acetate | 100 | 99 | 104 | 105 |
| 2-Butanol | 100 | 107 | 105 | 105 |
| Isobutanol | 100 | 107 | 105 | 105 |
| 1-Butanol | 100 | 95 | 98 | 99 |
| 3-Methyl-1-Butanol | 100 | 108 | 95 | 99 |
| Amyl Alcohol | 100 | 100 | 101 | 102 |

SI Table 3: Measured pH of Liquid Hand Sanitizer Products


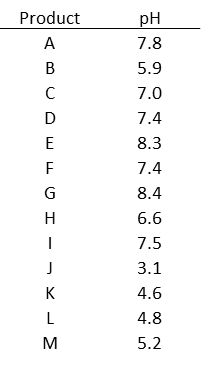


SI Table 4: Acetal Kinetics Study (% Acetal remaining)

The table reports the % acetal remaining over 6 h in the various buffers tested. Acetal displayed instability in pH 5 and pH 3.

| Time | Water | pH 8.5 | pH 6.6 | pH 5 | pH 3 |
| --- | --- | --- | --- | --- | --- |
| 0 h | 100 | 100 | 100 | 100 | 100 |
| 1.66 h | 97 | 98 | 100 | 93 | 6 |
| 3.33 h | 97 | 96 | 97 | 87 | 0 |
| 6 h | 96 | 94 | 95 | 77 | 0 |

SI Table 5: Acetaldehyde Kinetics Study (% Acetaldehyde remaining)

The table reports the % acetaldehyde remaining over 6 h in the various buffers tested. Acetaldehyde displayed stability in all buffers tested.

| Time | Water | pH 8.5 | pH 6.6 | pH 5 | pH 3 |
| --- | --- | --- | --- | --- | --- |
| 0 h | 100 | 100 | 100 | 100 | 100 |
| 1.66 h | 100 | 102 | 102 | 100 | 104 |
| 3.33 h | 100 | 101 | 103 | 100 | 104 |
| 6 h | 100 | 102 | 103 | 100 | 106 |

SI Table 6: Ethanol Kinetics Study (% Ethanol remaining)

The table reports the % ethanol remaining over 6 h in various buffers tested. Ethanol displayed stability in all buffers tested.

| Time | Water | pH 8.5 | pH 6.6 | pH 5 | pH 3 |
| --- | --- | --- | --- | --- | --- |
| 0 h | 100 | 100 | 100 | 100 | 100 |
| 1.66 h | 100 | 102 | 99 | 98 | 97 |
| 3.33 h | 98 | 100 | 96 | 96 | 97 |
| 6 h | 98 | 101 | 99 | 97 | 97 |

SI Table 7: Spike Recovery after pH Neutralization of Acidic Liquid Hand Sanitizer Product

The recovery of acetaldehyde and acetal is improved after the pH of Product K is adjusted to pH 6 using ammonium hydroxide.

|  | DMSO Control | Product K pH 4.6 | Product K adjusted pH 6.0 |
| --- | --- | --- | --- |
| Acetaldehyde | 100 | 144 | 78 |
| Methanol | 100 | 104 | 103 |
| Benzene | 100 | 105 | 104 |
| Acetal | 100 | 38 | 89 |

SI Table 8: Intraday (n=3) and interday (n=9) accuracy of quality control standards

|  |  | QC Standards | | | |
| --- | --- | --- | --- | --- | --- |
|  |  | LLOQ | Low | Mid | High |
| Ethanol | Day 1 | 100.5 | 104.9 | 105.8 | 101.0 |
|  | Day 2 | 100.9 | 108.5 | 105.8 | 100.6 |
|  | Day 3 | 92.4 | 103.8 | 103.7 | 99.1 |
|  | Interday | 97.9 | 105.7 | 105.1 | 100.2 |
| Isopropanol | Day 1 | 94.1 | 108.7 | 109.5 | 100.8 |
|  | Day 2 | 94.5 | 112.0 | 108.9 | 100.2 |
|  | Day 3 | 87.1 | 105.6 | 105.9 | 98.8 |
|  | Interday | 91.9 | 108.8 | 108.1 | 99.9 |
| Benzene | Day 1 | 100.5 | 101.7 | 101.4 | 100.5 |
|  | Day 2 | 108.1 | 105.1 | 101.2 | 99.5 |
|  | Day 3 | 95.1 | 101.9 | 98.2 | 99.0 |
|  | Interday | 101.2 | 102.9 | 100.3 | 99.7 |
| Acetaldehyde | Day 1 | 104.7 | 101.2 | 101.9 | 94.3 |
|  | Day 2 | 109.5 | 102.3 | 101.2 | 92.6 |
|  | Day 3 | 97.8 | 100.2 | 96.7 | 96.4 |
|  | Interday | 104.0 | 101.2 | 99.9 | 94.4 |
| Acetal | Day 1 | 111.4 | 96.7 | 97.0 | 97.6 |
|  | Day 2 | 114.9 | 98.7 | 96.4 | 97.2 |
|  | Day 3 | 105.1 | 94.4 | 94.1 | 95.3 |
|  | Interday | 110.5 | 96.6 | 95.8 | 96.7 |
| Methanol | Day 1 | 108.9 | 101.9 | 101.5 | 101.1 |
|  | Day 2 | 110.4 | 104.9 | 101.5 | 101.4 |
|  | Day 3 | 99.8 | 100.8 | 99.6 | 99.5 |
|  | Interday | 106.3 | 102.5 | 100.9 | 100.7 |

SI Table 9: Intraday (n=3) and interday (n=9) precision of quality control standards

|  |  | QC Standards | | | |
| --- | --- | --- | --- | --- | --- |
|  |  | LLOQ | Low | Mid | High |
| Ethanol | Day 1 | 3.11 | 3.01 | 1.05 | 0.87 |
|  | Day 2 | 0.55 | 0.67 | 1.28 | 0.26 |
|  | Day 3 | 1.38 | 1.32 | 1.21 | 3.52 |
|  | Interday | 4.61 | 2.60 | 1.45 | 2.00 |
| Isopropanol | Day 1 | 3.84 | 3.07 | 1.14 | 1.21 |
|  | Day 2 | 0.92 | 0.81 | 0.96 | 0.21 |
|  | Day 3 | 1.55 | 1.35 | 0.95 | 3.29 |
|  | Interday | 4.48 | 3.07 | 1.75 | 1.96 |
| Benzene | Day 1 | 3.70 | 1.63 | 1.65 | 0.50 |
|  | Day 2 | 1.74 | 1.55 | 0.98 | 0.24 |
|  | Day 3 | 4.55 | 3.38 | 1.44 | 1.70 |
|  | Interday | 6.33 | 2.59 | 1.95 | 1.10 |
| Acetaldehyde | Day 1 | 3.62 | 1.68 | 1.89 | 0.60 |
|  | Day 2 | 2.61 | 0.85 | 0.52 | 1.22 |
|  | Day 3 | 0.83 | 2.16 | 0.58 | 0.49 |
|  | Interday | 5.39 | 1.70 | 2.64 | 1.89 |
| Acetal | Day 1 | 2.21 | 1.96 | 1.31 | 0.25 |
|  | Day 2 | 1.83 | 2.83 | 0.76 | 1.20 |
|  | Day 3 | 0.50 | 1.37 | 0.53 | 2.74 |
|  | Interday | 4.18 | 2.70 | 1.60 | 1.86 |
| Methanol | Day 1 | 1.98 | 2.91 | 1.32 | 0.97 |
|  | Day 2 | 0.26 | 0.11 | 0.95 | 0.64 |
|  | Day 3 | 1.15 | 1.31 | 0.96 | 3.10 |
|  | Interday | 4.80 | 2.37 | 1.32 | 1.86 |

SI Table 10: Signal-to-Noise at LLOQ

∞ = infinity


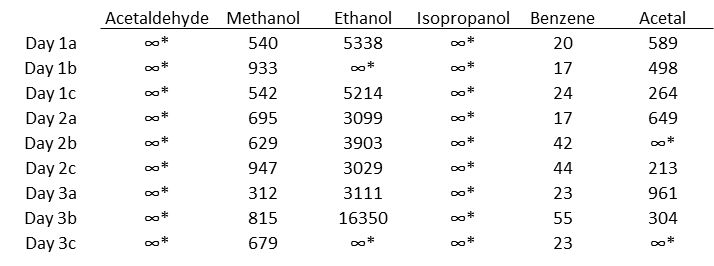


SI Table 11: Robustness Results

| **Ethanol** |  |  |  |  |  |  |  |
| --- | --- | --- | --- | --- | --- | --- | --- |
| Parameters | Change | Retention time | Area | USP tailing | Resolution | Capacity factor | USP theoretical plates |
| Nominal |  | 1.82 +/- 0.000 | 1059117 +/- 12500 | 0.86 +/- 0.009 | >2 | 1.30 +/- 0.000 | 1672 +/- 6.24 |
| Split ratio | (Low) 95:1 | 1.81 +/- 0.000 | 1127463 +/- 3150 | 1.34 +/- 0.004 | >2 | 1.28 +/- 0.000 | 1199 +/- 11.6 |
|  | (High) 105:1 | 1.82 +/- 0.000 | 1041951 +/- 14300 | 1.13 +/- 0.013 | >2 | 1.30 +/- 0.000 | 1758 +/- 28.2 |
| Equilibration, min | (Low) 9.5 | 1.82 +/- 0.000 | 1086558 +/- 15700 | 0.85 +/- 0.006 | >2 | 1.30 +/- 0.000 | 1683 +/- 7.37 |
|  | (High) 10.5 | 1.82 +/- 0.000 | 1057330 +/- 30000 | 0.84 +/- 0.004 | >2 | 1.30 +/- 0.000 | 1671 +/- 2.52 |
| Equilibration, C | (Low) 120 C | 1.82 +/- 0.000 | 941290 +/- 8250 | 0.84 +/- 0.010 | >2 | 1.30 +/- 0.000 | 1678 +/- 9.29 |
|  | (High) 130 C | 1.82 +/- 0.000 | 1223624 +/- 18500 | 0.83 +/- 0.008 | >2 | 1.30 +/- 0.000 | 1663 +/- 6.66 |
| GC Oven, C | (Low) | 1.89 +/- 0.000 | 1074310 +/- 51200 | 0.95 +/- 0.010 | >2 | 1.38 +/- 0.000 | 2150 +/- 89.5 |
|  | (High) | 1.76 +/- 0.000 | 1081539 +/- 11500 | 1.15 +/- 0.025 | >2 | 1.21 +/- 0.000 | 1550 +/- 5.69 |
|  |  |  |  |  |  |  |  |
| **Isopropanol** |  |  |  |  |  |  |  |
| Parameters | Change | Retention time | Area | USP tailing | Resolution | Capacity factor | USP theoretical plates |
| Nominal |  | 2.27 +/- 0.000 | 4021642 +/- 45800 | 0.95 +/- 0.007 | >2 | 1.85 +/- 0.000 | 2090 +/- 0.58 |
| Split ratio | (Low) 95:1 | 2.26 +/- 0.000 | 4267769 +/- 7240 | 1.30 +/- 0.001 | >2 | 1.84 +/- 0.000 | 1461 +/- 5.86 |
|  | (High) 105:1 | 2.27 +/- 0.000 | 3947313 +/- 56700 | 1.12 +/- 0.009 | >2 | 1.85 +/- 0.000 | 1364 +/- 0.00 |
| Equilibration, min | (Low) 9.5 | 2.27 +/- 0.000 | 4117059 +/- 64400 | 0.94 +/- 0.007 | >2 | 1.85 +/- 0.000 | 2091 +/- 0.00 |
|  | (High) 10.5 | 2.27 +/- 0.000 | 4010858 +/- 118000 | 0.93 +/- 0.002 | >2 | 1.85 +/- 0.000 | 2091 +/- 0.00 |
| Equilibration, C | (Low) 120 C | 2.27 +/- 0.000 | 3585173 +/- 31900 | 0.93 +/- 0.002 | >2 | 1.85 +/- 0.000 | 2091 +/- 0.00 |
|  | (High) 130 C | 2.27 +/- 0.000 | 4613801 +/- 70300 | 0.92 +/- 0.006 | >2 | 1.85 +/- 0.000 | 2091 +/- 0.00 |
| GC Oven, C | (Low) | 2.35 +/- 0.000 | 4078284 +/- 209000 | 1.30 +/- 0.019 | >2 | 1.96 +/- 0.000 | 2386 +/- 117 |
|  | (High) | 2.17 +/- 0.000 | 4082442 +/- 41800 | 1.34 +/- 0.063 | >2 | 1.74 +/- 0.000 | 1279 +/- 2.52 |
|  |  |  |  |  |  |  |  |
| **Benzene** |  |  |  |  |  |  |  |
| Parameters | Change | Retention time | Area | USP tailing | Resolution | Capacity factor | USP theoretical plates |
| Nominal |  | 4.13 +/- 0.000 | 2017 +/- 27 | 0.94 +/- 0.002 | >2 | 4.20 +/- 0.000 | 19356 +/- 3230 |
| Split ratio | (Low) 95:1 | 4.13 +/- 0.000 | 2170 +/- 36 | 0.75 +/- 0.007 | >2 | 4.20 +/- 0.000 | 19537 +/- 8680 |
|  | (High) 105:1 | 4.13 +/- 0.000 | 1943 +/- 30 | 1.21 +/- 0.004 | >2 | 4.20 +/- 0.000 | 21110 +/- 8260 |
| Equilibration, min | (Low) 9.5 | 4.13 +/- 0.000 | 2032 +/- 16 | 0.94 +/- 0.003 | >2 | 4.20 +/- 0.000 | 22586 +/- 4480 |
|  | (High) 10.5 | 4.13 +/- 0.000 | 1990 +/- 69 | 0.91 +/- 0.005 | >2 | 4.20 +/- 0.000 | 16773 +/- 55400 |
| Equilibration, C | (Low) 120 C | 4.13 +/- 0.000 | 1825 +/- 27 | 0.93 +/- 0.040 | >2 | 4.20 +/- 0.000 | 17467 +/- 5700 |
|  | (High) 130 C | 4.13 +/- 0.000 | 2201 +/- 48 | 0.93 +/- 0.15 | >2 | 4.20 +/- 0.000 | 19495 +/- 8390 |
| GC Oven, C | (Low) | 4.23 +/- 0.000 | 1954 +/- 69 | 1.20 +/- 0.015 | >2 | 4.33 +/- 0.000 | 28544 +/- 6780 |
|  | (High) | 4.02 +/- 0.000 | 2058 +/- 36 | 1.30 +/- 0.024 | >2 | 4.05 +/- 0.000 | 14016 +/- 8240 |
|  |  |  |  |  |  |  |  |
| **Acetaldehyde** |  |  |  |  |  |  |  |
| Parameters | Change | Retention time | Area | USP tailing | Resolution | Capacity factor | USP theoretical plates |
| Nominal |  | 1.28 +/- 0.000 | 4885 +/- 164 | 1.08 +/- 0.013 | >2 | 0.61 +/- 0.000 | 2547 +/- 917 |
| Split ratio | (Low) 95:1 | 1.28 +/- 0.000 | 5201 +/- 121 | 0.95 +/- 0.002 | >2 | 0.61 +/- 0.000 | 1962 +/- 356 |
|  | (High) 105:1 | 1.28 +/- 0.000 | 4696 +/- 40 | 1.22 +/- 0.010 | >2 | 0.61 +/- 0.000 | 3728 +/- 152 |
| Equilibration, min | (Low) 9.5 | 1.28 +/- 0.000 | 4818 +/- 60 | 1.07 +/- 0.009 | >2 | 0.61 +/- 0.000 | 2142 +/- 289 |
|  | (High) 10.5 | 1.28 +/- 0.000 | 4713+/- 88 | 1.06 +/- 0.003 | >2 | 0.61 +/- 0.000 | 2041 +/- 172 |
| Equilibration, C | (Low) 120 C | 1.28 +/- 0.000 | 4300 +/- 49 | 1.07 +/- 0.006 | >2 | 0.61 +/- 0.000 | 2019 +/- 128 |
|  | (High) 130 C | 1.28 +/- 0.000 | 4977 +/- 12 | 1.07 +/- 0.013 | >2 | 0.61 +/- 0.000 | 2316 +/- 642 |
| GC Oven, C | (Low) | 1.30 +/- 0.005 | 4586 +/- 16 | 1.21 +/- 0.412 | >2 | 0.63 +/- 0.007 | 2886 +/- 218 |
|  | (High) | 1.26 +/- 0.000 | 4570 +/- 83 | 0.92 +/- 0.009 | >2 | 0.58 +/- 0.000 | 2630 +/- 906 |
|  |  |  |  |  |  |  |  |
| **Acetal** |  |  |  |  |  |  |  |
| Parameters | Change | Retention time | Area | USP tailing | Resolution | Capacity factor | USP theoretical plates |
| Nominal |  | 4.89 +/- 0.005 | 18079 +/- 280 | 0.98 +/- 0.304 | >2 | 5.15 +/- 0.006 | 13193 +/- 1420 |
| Split ratio | (Low) 95:1 | 4.88 +/- 0.000 | 19495 +/- 82 | 1.13 +/- 0.005 | >2 | 5.15 +/- 0.000 | 9145 +/- 1280 |
|  | (High) 105:1 | 4.89 +/- 0.000 | 17523 +/- 30 | 0.87 +/- 0.004 | >2 | 5.15 +/- 0.000 | 13858 +/- 1910 |
| Equilibration, min | (Low) 9.5 | 4.88 +/- 0.000 | 18534 +/- 451 | 1.32 +/- 0.016 | >2 | 5.14 +/- 0.000 | 10298 +/- 1100 |
|  | (High) 10.5 | 4.88 +/- 0.004 | 18001 +/- 609 | 1.15 +/- 0.287 | >2 | 5.14 +/- 0.005 | 12512 +/- 969 |
| Equilibration, C | (Low) 120 C | 4.89 +/- 0.001 | 16704 +/- 170 | 0.80 +/- 0.026 | >2 | 5.15 +/- 0.001 | 13568 +/- 110 |
|  | (High) 130 C | 4.88 +/- 0.000 | 19819 +/- 311 | 1.28 +/- 0.006 | >2 | 5.14 +/- 0.000 | 9804 +/- 616 |
| GC Oven, C | (Low) | 4.99 +/- 0.001 | 18211 +/- 1140 | 1.25 +/- 0.042 | >2 | 5.28 +/- 0.001 | 19178 +/- 4120 |
|  | (High) | 4.78 +/- 0.005 | 18593 +/- 448 | 0.98 +/- 0.272 | >2 | 5.01 +/- 0.006 | 10238 +/- 2250 |
|  |  |  |  |  |  |  |  |
| **Methanol** |  |  |  |  |  |  |  |
| Parameters | Change | Retention time | Area | USP tailing | Resolution | Capacity factor | USP theoretical plates |
| Nominal |  | 1.34 +/- 0.000 | 49736 +/- 786 | 0.93 +/- 0.007 | >2 | 0.69 +/- 0.000 | 1010 +/- 0.58 |
| Split ratio | (Low) 95:1 | 1.33 +/- 0.000 | 53368 +/- 206 | 1.55 +/- 0.010 | >2 | 0.68 +/- 0.000 | 603 +/- 1.15 |
|  | (High) 105:1 | 1.34 +/- 0.000 | 48910 +/- 666 | 1.04 +/- 0.007 | >2 | 0.69 +/- 0.000 | 1027 +/- 23.5 |
| Equilibration, min | (Low) 9.5 | 1.34 +/- 0.000 | 51098 +/- 689 | 0.93 +/- 0.006 | >2 | 0.69 +/- 0.000 | 1006 +/- 2.65 |
|  | (High) 10.5 | 1.34 +/- 0.000 | 49569 +/- 137 | 0.92 +/- 0.004 | >2 | 0.69 +/- 0.000 | 1003 +/- 4.62 |
| Equilibration, C | (Low) 120 C | 1.34 +/- 0.000 | 43790 +/- 396 | 0.93 +/- 0.008 | >2 | 0.69 +/- 0.000 | 1001 +/- 6.24 |
|  | (High) 130 C | 1.34 +/- 0.000 | 58045 +/- 941 | 0.91 +/- 0.008 | >2 | 0.69 +/- 0.000 | 1002 +/- 5.20 |
| GC Oven, C | (Low) | 1.37 +/- 0.000 | 50214 +/- 244 | 1.03 +/- 0.007 | >2 | 0.72 +/- 0.000 | 1069 +/- 5.57 |
|  | (High) | 1.31 +/- 0.000 | 50809 +/- 602 | 0.91 +/- 0.012 | >2 | 0.65 +/- 0.000 | 961 +/- 1.00 |

SI Table 12: Stock solution stability, 96 h

|  | Validation QC Concentrations (ug/mL) | | | |
| --- | --- | --- | --- | --- |
|  | LLOQ | Low | Mid | High |
| Acetaldehyde | 104 | 104 | 105 | 96 |
| Methanol | 105 | 106 | 107 | 104 |
| Ethanol | 104 | 105 | 106 | 104 |
| Isopropanol | 104 | 105 | 107 | 104 |
| Benzene | 103 | 103 | 104 | 99 |
| Acetal | 100 | 102 | 104 | 100 |
| Acetone-d6 | 102 | 102 | 101 | 102 |
| Cyclohexane | 93 | 99 | 103 | 95 |

SI Table 13: Autosampler stability in DMSO, 72 h

|  | Validation QC Concentrations (ug/mL) | | | |
| --- | --- | --- | --- | --- |
|  | LLOQ | Low | Mid | High |
| Acetaldehyde | 107 | 103 | 104 | 102 |
| Methanol | 107 | 104 | 104 | 102 |
| Ethanol | 105 | 103 | 101 | 98 |
| Isopropanol | 106 | 104 | 103 | 101 |
| Benzene | 104 | 101 | 101 | 98 |
| Acetal | 108 | 104 | 104 | 102 |
| Acetone-d6 | 100 | 103 | 103 | 106 |
| Cyclohexane | 98 | 101 | 102 | 104 |

SI Table 14: Autosampler Stability in Hand Sanitizer Products, QC LLOQ (25% of NMT Limit), 72 h

|  | Product A | Product B | Product C | Product D |
| --- | --- | --- | --- | --- |
| Acetaldehyde | 105 | 104 | 88 | 92 |
| Methanol | 102 | 99 | 89 | 92 |
| Ethanol | 101 | 100 | 90 | 92 |
| Isopropanol | 102 | 98 | 100 | 101 |
| Benzene | 104 | 92 | 96 | 94 |
| Acetal | 103 | 103 | 90 | 93 |
| Acetone-d6 | 101 | 101 | 93 | 94 |
| Cyclohexane | 100 | 100 | 94 | 95 |

SI Table 15: Autosampler Stability in Hand Sanitizer Products, Spiking Solution (100% of NMT Limit), 72 h

|  | Product A | Product B | Product C | Product D |
| --- | --- | --- | --- | --- |
| Acetaldehyde | 104 | 102 | 91 | 92 |
| Methanol | 101 | 99 | 91 | 92 |
| Ethanol | 101 | 100 | 92 | 94 |
| Acetone | 101 | 99 | 93 | 94 |
| Isopropanol | 102 | 99 | 101 | 101 |
| 1-Propanol | 103 | 99 | 92 | 94 |
| Ethyl Acetate | 102 | 99 | 93 | 95 |
| 2-Butanol | 103 | 99 | 94 | 95 |
| Isobutanol | 104 | 100 | 94 | 95 |
| Benzene | 101 | 97 | 90 | 94 |
| 1-Butanol | 103 | 96 | 92 | 94 |
| Acetal | 104 | 103 | 92 | 93 |
| 3-Methyl-1-Butanol | 104 | 99 | 94 | 95 |
| Amyl Alcohol | 103 | 97 | 95 | 96 |
| Acetone-d6 | 101 | 101 | 93 | 94 |
| Cyclohexane | 101 | 100 | 94 | 95 |

SI Table 16: Autosampler Stability in Hand Sanitizer Products, QC High (300% of NMT Limit), 72 h

|  | Product A | Product B | Product C | Product D |
| --- | --- | --- | --- | --- |
| Acetaldehyde | 101 | 100 | 91 | 91 |
| Methanol | 105 | 98 | 91 | 92 |
| Ethanol | 100 | 100 | 93 | 94 |
| Isopropanol | 102 | 99 | 100 | 100 |
| Benzene | 103 | 98 | 93 | 94 |
| Acetal | 96 | 101 | 92 | 91 |
| Acetone-d6 | 98 | 100 | 93 | 94 |
| Cyclohexane | 104 | 99 | 93 | 94 |

SI Table 17: Dilution Integrity

|  | Expected Concentration  (µg/mL) | Calculated Concentration  (µg/mL) | Accuracy  (%) | Precision  (%RSD) |
| --- | --- | --- | --- | --- |
| Ethanol | 552.3 | 595.7 | 107.9 | 1.93 |
| Isopropanol | 550.2 | 584.9 | 106.3 | 2.39 |

SI Table 18: Check QC standards to monitor instrument performance at QC LLOQ and QC High for accuracy and precision during product testing

Check QC standards were prepared at QC LLOQ (25% of the impurity limit) and at QC High (300% of the impurity limit) to span the range of the analytical method. The check QC standards were injected during the analysis day for method application with n=3 samples injected before hand sanitizer products (set #1), n=3 samples injected in the middle of hand sanitizer product testing (set #2), and n=3 samples injected after the hand sanitizer products (set #3). The triplicate sets of check QC standards were analyzed individually (n=3) and collectively (n=9) to evaluate the accuracy and precision of the method during product testing.

|  | Set #1 (n=3) | | Set #2 (n=3) | | Set #3 (n=3) | | Combined (n=9) | |
| --- | --- | --- | --- | --- | --- | --- | --- | --- |
| LOQ (25%) | Accuracy | Precision | Accuracy | Precision | Accuracy | Precision | Accuracy | Precision |
| Acetaldehyde | 104.6 | 1.6 | 102.9 | 1.6 | 101.4 | 0.6 | 103.0 | 1.8 |
| Methanol | 104.9 | 1.9 | 104.4 | 1.4 | 104.0 | 1.4 | 104.5 | 1.4 |
| Ethanol | 96.6 | 1.6 | 97.6 | 1.3 | 97.1 | 1.5 | 97.1 | 1.3 |
| Isopropanol | 90.5 | 1.7 | 91.0 | 1.8 | 91.0 | 1.2 | 90.8 | 1.4 |
| Benzene | 105.7 | 5.7 | 108.4 | 3.2 | 103.2 | 3.8 | 105.8 | 4.3 |
| Acetal | 108.7 | 0.3 | 110.0 | 1.4 | 109.9 | 0.2 | 109.6 | 0.9 |
|  |  |  |  |  |  |  |  |  |
|  | Set #1 (n=3) | | Set #2 (n=3) | | Set #3 (n=3) | | Combined (n=9) | |
| High (300%) | Accuracy | Precision | Accuracy | Precision | Accuracy | Precision | Accuracy | Precision |
| Acetaldehyde | 98.5 | 4.1 | 96.6 | 2.7 | 95.5 | 0.6 | 96.9 | 2.8 |
| Methanol | 100.0 | 3.6 | 99.7 | 2.4 | 99.7 | 0.3 | 99.8 | 2.2 |
| Ethanol | 100.2 | 3.3 | 100.3 | 2.8 | 100.3 | 0.7 | 100.3 | 2.2 |
| Isopropanol | 100.0 | 2.6 | 100.4 | 2.5 | 101.6 | 0.4 | 100.7 | 1.9 |
| Benzene | 99.2 | 4.5 | 100.8 | 2.9 | 100.0 | 0.5 | 100.0 | 2.8 |
| Acetal | 98.2 | 5.1 | 102.0 | 3.3 | 100.6 | 0.7 | 100.3 | 3.5 |

SI Table 19: Spike Recovery Results During Method Application

Products B-D were used during method validation only and not for method application testing. The determined active ingredient is listed for each product and the spike recovery accuracy (%) for each impurity is provided in the table.

|  |  | Product A | Product E | Product F | Product G | Product H | Product I |
| --- | --- | --- | --- | --- | --- | --- | --- |
| Limit (ppm) |  | Ethanol | Ethanol | Isopropanol | Isopropanol | Isopropanol | Isopropanol |
| 2 | Benzene | 96 | 98 | 104 | 100 | 105 | 105 |
| 50 | Acetaldehyde | 91 | 96 | 98 | 99 | 96 | 97 |
| 50 | Acetal | 104 | 98 | 105 | 103 | 106 | 107 |
| 630 | Methanol | 94 | 94 | 99 | 97 | 99 | 98 |
| 1000 | 1-Propanol | 103 | 109 | 105 | 104 | 109 | 107 |
| 1000 | 1-Butanol | 106 | 108 | 104 | 104 | 108 | 106 |
| 2200 | Ethyl Acetate | 101 | 102 | 99 | 100 | 104 | 102 |
| 4100 | 3-Methyl-1-Butanol | 102 | 103 | 103 | 102 | 107 | 105 |
| 4100 | Amyl Alcohol | 101 | 100 | 101 | 101 | 105 | 104 |
| 4400 | Acetone | 98 | 97 | 98 | 98 | 98 | 99 |
| 6200 | 2-Butanol | 104 | 106 | 102 | 102 | 108 | 106 |
| 21700 | Isobutanol | 102 | 105 | 100 | 100 | 105 | 103 |
